# Resistance of *Staphylococcus aureus* to antimicrobial agents in Ethiopia: a meta-analysis

**DOI:** 10.1186/s13756-017-0243-7

**Published:** 2017-08-23

**Authors:** Serawit Deyno, Sintayehu Fekadu, Ayalew Astatkie

**Affiliations:** 1 Department of Pharmacology, School of Medicine, College of Medicine and Health Sciences, Hawassa University, P. O. Box 1560, Hawassa, Ethiopia; 2 Department of Microbiology, Faculty of Medicine, Shimane University, Shimane, Japan; 3 School of Public and Environmental Health, College of Medicine and Health Sciences, Hawassa University, Hawassa, Ethiopia

**Keywords:** Antimicrobial resistance, *Staphylococcus aureus*, Meta-analysis, Ethiopia

## Abstract

**Background:**

Emergence of antimicrobial resistance by *Staphylococcus aureus* has limited treatment options against its infections. The purpose of this study was to determine the pooled prevalence of resistance to different antimicrobial agents by *S. aureus* in Ethiopia.

**Methods:**

Web-based search was conducted in the databases of PubMed, Google Scholar, Hinari, Scopus and the Directory of Open Access Journals (DOAJ) to identify potentially eligible published studies. Required data were extracted and entered into Excel spread sheet. Statistical analyses were performed using Stata version 13.0. The *metaprop* Stata command was used to pool prevalence values. Twenty-one separate meta-analysis were done to estimate the pooled prevalence of the resistance of *S. aureus* to twenty-one different antimicrobial agents. Heterogeneity among the studies was assessed using the I^2^ statistic and chi-square test. Publication bias was assessed using Egger’s test. Because of significant heterogeneity amongst the studies, the random effects model was used to pool prevalence values.

**Results:**

The electronic database search yielded 1317 studies among which 45 studies met our inclusion criteria. Our analyses demonstrated very high level of resistance to amoxicillin (77% [95% confidence interval (CI): 68%, 0.85%]), penicillin (76% [95% CI: 67%, 84%]), ampicillin (75% [95% CI: 65%, 85%]), tetracycline (62% [95% CI: 55%, 68%]), methicillin (47% [95% CI: 33%, 61%]), cotrimoxaziole (47% [95% CI: 40%, 55%]), doxycycline (43% [95% CI: 26%, 60%]), and erythromycin (41% [95% CI: 29%, 54%]). Relatively low prevalence of resistance was observed with kanamycin (14% [95% CI: 5%, 25%]) and ciprofloxacin (19% [95% CI: 13%, 26%]). The resistance level to vancomycin is 11% 995% CI: (4%, 20%). High heterogeneity was observed for each of the meta-analysis performed (I^2^ ranging from 79.36% to 95.93%; all *p*-values ≤0.01). Eggers’ test did not show a significant publication bias for all antimicrobial agents except for erythromycin and ampicillin.

**Conclusions:**

*S. aureus* in Ethiopia has gotten notoriously resistant to almost to all of antimicrobial agents in use including, penicillin, cephalosporins, tetracyclines, chloramphenicol, methicillin, vancomycin and sulphonamides. The resistance level to vancomycin is bothersome and requires a due attention. Continued and multidimensional efforts of antimicrobial stewardship program promoting rational use of antibiotics, infection prevention and containment of AMR are urgently needed.

**Electronic supplementary material:**

The online version of this article (doi:10.1186/s13756-017-0243-7) contains supplementary material, which is available to authorized users.

## Background

*Staphylococcus aureus* (*S. aureus*) infection is a major cause of skin, soft tissue, respiratory, bone, joint, and cardiovascular disorders [1]. *S. aureus* remains a versatile and dangerous pathogen in humans. The frequencies of both community-acquired and hospital-acquired staphylococcal infections have increased steadily. Treatment of these infections has become more difficult because of the emergence of multidrug-resistant strains [2].

Various mechanisms are responsible for *S. aureus* antimicrobial resistance (AMR). Penicillin is inactivated by β-lactamase. AMR to methicillin confers resistance to all β-lactamase-resistant penicillin’s and cephalosporins which require the presence of the mec gene that encodes penicillin-binding protein [3]. The enterococcal plasmid-bearing gene for resistance to vancomycin has been transferred by conjugation to *S. aureus* in vitro [4]. Both increased cell-wall synthesis and alterations in the cell wall that prevent vancomycin from reaching sites of cell-wall synthesis have been suggested as mechanisms [4]. Increase in vancomycin use has led to the emergence of two types of glycopeptide-resistant *S. aureus*. The first one, designated vancomycin intermediate-resistant *S. aureus* (VISA), is associated with a thickened and poorly cross-linked cell wall is due to continuous exposure to glycopeptide. The second type, vancomycin-resistant *S. aureus* (VRSA), is due to acquisition from *Enterococcus* species of the *vanA* operon resulting in high-level resistance and is a rare phenomenon [5].

In Ethiopia the first published antimicrobial preliminary report on AMR was published by Plorde et al. in 1970 for different microbial agents [6]. Beginning from that time AMR report were made by different antimicrobial surveillances and studies, it showed rapid rise and spread of resistant strains.

Facilitating more appropriate choices of treatment, minimizing the morbidity and mortality due to resistant infections, and preserving the effectiveness of antimicrobials requires summarization and synthesis of the evidence regarding AMR in a country. Appropriately summarized and synthesized evidence is mandatory for updating national treatment guidelines. To our knowledge, no previous meta-analysis or systematic review has been conducted on *S. aureus* AMR to all antimicrobial commonly in use in Ethiopia. The purpose of this study was, therefore, to determine pooled prevalence of *S. aureus* resistance to common antimicrobial agents in Ethiopia based on the best available studies.

## Methods

### Study design

This study did a meta-analysis of prevalence of *S. aures* resistance to different antimicrobial agents in Ethiopia using the best available studies.

### Literature search strategy

Web-based search using PubMed, Google Scholar, Hinari, Scopus and the Directory of Open Access Journals (DOAJ) was conducted in June 2016. Google search was used for unpublished works and government documents. Two of the authors (SD and SF) independently searched for relevant studies to be included in this meta-analysis. The PubMed search was carried out via the EndNote software. Relevant search results from Google scholar, Embase, Scopus and the DOAJ were individually downloaded and manually entered into EndNote. The reference lists of the identified studies were used to identify other relevant studies.

The search was done using various key words: Staphylococcus, antimicrobial resistance, antibiotic resistance, drug resistance, drug susceptibility, antibacterial resistance, Ethiopia. These key terms were used in various combinations using Boolean search technique. We did not limit the search by year or language of publication.

### Study selection procedures and criteria

Study selection was performed in two stages independently by two of the authors (SD and SF). First, the titles and abstracts of all retrieved articles were reviewed and then grouped as “eligible for inclusion” if they did address the study question and “ineligible for inclusion” if they did not. Second, articles which were grouped under “eligible for inclusion” were reviewed in full detail for decision.

All available studies and data were included based on the following predefined inclusion criteria. 1) Studies that were original journal articles, short communications, or unpublished works; 2) Studies that did the antimicrobial susceptibility test according to the criteria of the Clinical Laboratory Standards Institute (CLSI) and defined antimicrobial resistance range according to CLSI manual [7], 3) Studies which used human infection sample.

Studies that 1) were duplicates, 2) were based on small number of isolates (1–10), 3) were conducted on non-human samples like on foods, food handlers’ belongings, health workers belongings or health workers carriage and 4) which were based on non-infectious carriage were excluded from this meta-analysis.

### Data extraction

Required data were extracted from eligible studies using Excel spreadsheet format prepared for this purpose by AA and SD. The data extracted from eligible studies include name of author(s), year of publication, place where the study was conducted, study design, total number of *S. aureus* isolate tested in the study, number of resistant *S. aureus* isolates, and isolate source. If the proportion of drug sensitive isolates (q) was reported, the number of resistant isolates was calculated by multiplying the number of isolates (n) by one minus the proportion of drug sensitive isolates (1-q) and if the proportion of drug resistant isolates was given the number of resistant isolates was found by multiplying the proportion (p) with total number of isolates (n).

### Statistical analysis and reporting

Statistical analyses were performed using Stata version 13.0 (Statacorp, LP, college station, TX). The prevalence values from the different studies were pooled using the *metaprop* command in Stata [8]. We did twenty-one separate meta-analyses to estimate the pooled prevalence of the resistance of *S. aureus* to twenty one different antimicrobial agents. The number of studies included in each of the meta-analyses ranged from 4 to 39. Heterogeneity amongst the studies was assessed using the I^2^ statistic. Because of significant heterogeneity amongst the studies the random-effects model (REM) was used to estimate the pooled prevalence and 95% CIs using the DerSimonian and Laird method [9]. The Freeman-Tukey double arcsine transformation was used so that studies reporting proportions near or at 0 and 1 would not be excluded from the meta-analysis. The possible presence of publication bias was checked using Egger’s test [10].

For studies that appeared to report unusually higher prevalence of resistance compared to others, we did sensitivity analysis after dropping the study which we suspected of reporting a higher-than-usual result. If the point estimate of pooled prevalence after dropping a study lies within the 95% CI of the overall pooled estimate for all studies combined, we considered the given study as having non-significant influence on the overall pooled estimate. Otherwise, the study was considered as having significantly influencing the overall estimate.

Results of the current meta-analysis are reported as per the Preferred Reporting Items for Systematic Reviews and Meta-Analysis (PRISMA) guideline. The PRISMA checklist was used to ensure inclusion of relevant information (the filled checklist is included as Additional file 1: S1) [11].

## Results

### Included studies and characteristics

**Fig. 1:**
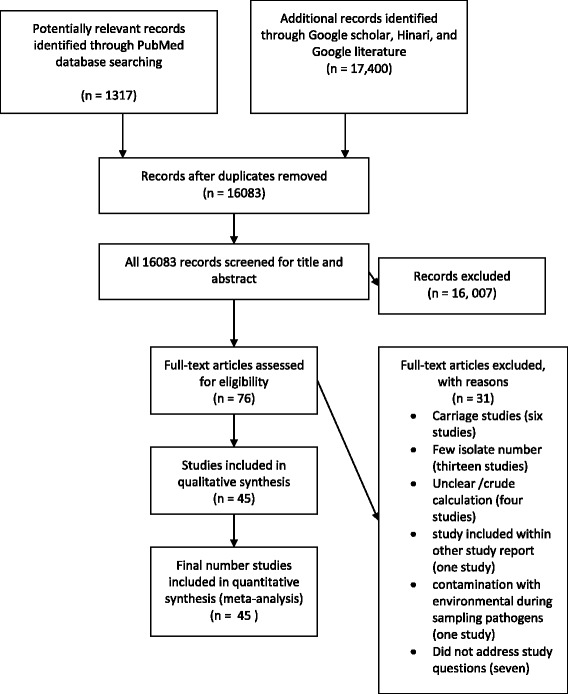
Flow diagram of retrieval of studies: Number of studies screened, assessed for eligibility, and included in the meta-analysis with reasons

The electronic database search yielded 1317 from PubMed and 17,400 from Google scholar, Hinari, and Google search of which 16,083 articles remained after removing duplicate articles. Title and abstract screening reduced eligible articles to 76 for full text evaluation. After reading the full texts, 31 studies were excluded for various reasons. Thirteen studies were excluded as their report is based on small number of isolates (less than or equal to10) [12–24], four studies reported crude resistance for all bacterial pathogen isolated [25–28], eleven did not address our study question [29–36], six studies were based on samples taken from of healthy carriers [37–42], one study [43] was part of another study [44], and one study [45] suffered from environmental contamination of the samples during processing. Thus, 45 studies met our inclusion criteria (Fig. 1). Forty-one of the studies were journal articles, three were unpublished works [46–48] and one was an official government document from the Drug Administration and Control Authority (DACA) of Ethiopia [49].

*S. aureus* isolates from a total of 4570 patients were tested for their antimicrobial resistance. The isolates were from ear discharge [50–57], eye discharge [47, 58–60], blood [61–68], wound infection [69–74], surgical site infection [30, 73, 75–78], mixed samples [6, 46, 48, 49, 79–83], leprosy ulcer [84, 85], and urine sample [86, 87]. Twenty nine studies used primary data while nineteen studies used records from hospitals or regional laboratories (the characteristics of each included study is summarized Table 1).

**Table 1:** Characteristics of included studies

No	Study	Study period	Sample source	Data type	Sample size	No of *S. aureus* isolate
1.	Hailu et al.*,* 2016	Jan.2013 -Apr. 2015.	Ear discharge	Secondary	368	78
2.	Abera et al.*,* 2008	Apr -June 2006	Different clinical samples	Primary	221	142
3.	Abera et al.*,* 2009	Sep. 2000 - Dec. 2008.	Ear discharge	Secondary	777	260
4.	Mama et al.*,* 2014	May toSep. 2013.	wound infection	Primary	150	47
5.	Shiferaw et al.*,* 2015	Feb.-May 2014	External ocular infections	Primary	160	21
6.	Sewunet, et al.*,* 2013	Apr-July 2010	Burn patients wound	Primary	50	24
7.	Alebachew et al.*,* 2012	March–May 2011	Wound burn patient’s	Primary	114	66
8.	Biadglegne, et al.*,* 2009	Sep. 2003-June 2008.	Urinary tract infections	Secondary	529	31
9.	Ferede et al.*,* 2001	2001	Ear swabs	Primary	112	28
10.	Guta et al.*,* 2014	Nov 2010-June 2011	surgical wounds	Primary	100	45
11.	Kahsay et al.*,* 2014	Dec 2011-Mar 2012	surgical wounds	Primary	184	73
12.	Mengesha et al.*,* 2014	Jan-June 2012	Surgical wounds	Primary	128	40
13.	Mulu et al.*,* 2012	Oct 2010-Jan2011	post-operative wounds	Primary	294	11
14.	Denboba et al.*,* 2016	2001–2011	Ear discharge	Secondary	1225	241
15.	Abera et al.*,* 2011	2003–2011	Ear discharge	Secondary	897	207
16.	Kibret et al.*,* 2010	2003 to 2010	Different clinical samples	Secondary	429	429
17.	Tenssay et al.*,* 2002	May 1997-Aug1998	Different Clinical samples	Primary	545	61
18.	Wasihun et al.*,* 2015	Nov 2014-June2015.	Ear discharege	Primary	162	46
19.	Worku et al.*,* 2014	June-Oct 2013	Ear discharge	Primary	117	24
20.	Negussie et al.*,* 2015	Oct 2011-Feb2012	Blood samples	Primary	201	13
21.	Gizachew et al.*,* 2015	Sep-Feb2013/2014	Different Clinical samples	Secondary	4321	309
22.	Yismaw et al.*,* 2008	Sep2001- Aug2005	Different Clinical samples	Secondary	-	616
23.	Ali et al.*,* 2008	Mar2001-apr2005	Blood sample	Secondary	472	34
24.	Mulu et al.*,* 2006	2005	wound infection	Secondary	151	51
25.	Azene et al.*,* 2011	2003 to 2010	wound infection	Secondary	599	208
26.	Dessalegn et al.*,* 2014	Nov2010 - Mar2011	post-surgical wound	Primary	194	66
27.	Gebrehiwot et al.*,* 2012	July 2011–July 2012	Blood sample	Primary	181	17
28.	Lemma et al.*,* 2012	Aug2006 – May 2007	leprosy ulcer	Primary	1827	68
29.	Shitaye et al.*,* 2010	Oct 2006 -Mar2007	Blood samples	Primary	302	17
30.	Alebachew et al. 2016	March–May, 2013	Blood samples	Primary	100	13
31.	Dagnew, et al. 2013	Sept 2006 to Jan 2012	Blood sample	Secondary	390	17
32.	Endris, et al. 2014	Feb - May, 2012.	Blood samples	Primary	83	11
33.	Godebo et al.*,* 2013	June-Dec., 2011	Wound	Primary	322	73
34.	Muluye et al.*,* 2013	2009–2012	Ear discharge	Secondary	250	54
35.	Muluye et al.*,* 2014	2009–2012	ocular infection	Secondary	102	13
36.	Wasihun et al.*,* 2015	Nov 2014–2015	Blood culture	Primary	514	54
37.	Ramos et al.*,* 2014	July–December, 2013	Sample from pus	Primary	68	15
38.	Dessie et al.*,* 2016	Oct2013-Mar2014	Wound	Primary	107	19
39.	Plorde et al.*,* 1970	Oct1969- Apr.1970	different clinical specimens	Primary	-	52
40.	Wolday et al.*,* 1997	Jan1992–1994	Urine sample	Secondary	672	16
41.	Tesfaye 2013	Feb2012-Oct2012.	external ocular infection	Primary	198	42
42.	DACA 2009	2004 to 2008	Different clinical samples	Secondary	1422	722
43.	Tadesse 2014 (UP^a^)	Dec 2013-Jun2014	Different clinical samples	Primary	188	79
44.	Neway et al.*,* 2006(UP^a^)	May-Aug. 2015	external ocular infection	Primary	288	63
45.	Endalafer 2008(UP^a^)	Jun2007-Apr2008	Different clinical samples	Primary	215	14

^a^UP = unpublished work

### Publication bias and heterogeneity

Evidence of high heterogeneity was observed for each of the meta-analyses performed (I^2^ ranging from 79.36% to 95.93%; all *p*-values ≤ 0.01). Eggers’ test did not suggest any significant publication bias except for erythromycin and ampicillin (see Additional file 2: S2).

### Prevalence of *S. aureus* resistance to different antimicrobial agents

Summary of the pooled prevalence of *S. aureus* AMR prevalence for twenty-one different antimicrobial agents and the number of studies included in the meta-analysis for each agent are presented in Table 2. Prevalence of *S. aureus* resistance for each antimicrobial agent based on pharmacological classification of the agents is given below. As new anti-MRSA agents such as linezolid, daptomycin, tigecycline, telavancin and ceftaroline are rarely available in Ethiopia and no published studies available on resistance to this agents, our results do not cover such agents.

**Table 2:** Pooled prevalence of *S. aureus* resistance to different antimicrobial agents in Ethiopia

	Agent	Number of studies	Total no of isolate tested	No of resistant isolate	Pooled AMR prevalence (95% CI)	I^2^ (*p*-value)
1.	Vancomycin	19	1750	132	0.11 (0.04,0.20)	95.34 (*P* ≤ 0.01)
2.	Methicilin	26	1179	843	0.47 (0.33,0.61)	96.82 (*P* ≤ 0.01)
3.	Ciprofloxacin	31	2254	400	0.19 (0.13, 0.26)	93.06(*P* ≤ 0.01)
4.	Tetracycline	36	3019	1982	0.62 (0.55, 0.68)	92.06(*P* ≤ 0.01)
5.	Cotrimoxazole	35	2825	1364	0.47 (0.40, 0.55)	92.85(*P* ≤ 0.01)
6.	Chloramphenicol	34	2763	1128	0.37 (0.29,0.45)	94.28 (*P* ≤ 0.01)
7.	Erythromycin	37	3828	2222	0.41 (0.29, 0.54)	98.28(*P* ≤ 0.01)
8.	Penicillin	33	2271	1627	0.76 (0.67, 0.84)	95.05(*P* ≤ 0.01)
9.	Clindamycin	14	1445	414	0.24 (0.12,0.37)	95.48(*P* ≤ 0.01)
10.	Carbencillin	6	398	184	0.34 (0.17,0.54)	86.89(*P* ≤ 0.01)
11.	Amoxicillin	18	870	660	0.77 (0.68,0.85)	87.44(*P* ≤ 0.01)
12.	Amoxicillin-clavulanic	12	524	166	0.30 (0.19,0.43)	88.64(*P* ≤ 0.01)
13.	Ampicillin	27	1814	1181	0.75 (0.65,0.85)	95.21(*P* ≤ 0.01)
14.	Gentamycin	39	3348	892	0.26 (0.18,0.34)	95.93(*P* ≤ 0.01)
15.	Ceftriaxone	28	2032	626	0.34 (0.25,0.43)	94.02(*P* ≤ 0.01)
16.	Cephalothin	15	2330	785	0.30 (0.18,0.43)	94.02(*P* ≤ 0.01)
17.	Cefoxitine	6	374	101	0.27 (0.06, 0.54)	95.74(*P* ≤ 0.01)
18.	Doxycline	14	541	239	0.43 (0.26,0.60)	93.32(*P* ≤ 0.01)
19.	Amikacin	4	211	62	0.23 (0.07,0.44)	90.42(*P* ≤ 0.01)
20.	Kanamycin	7	66	40	0.14 (0.05,0.25)	79.36(*P* ≤ 0.01)
21.	Norfloxacilin	11	751	186	0.25 (0.14,0.38)	92.79(*P* ≤ 0.01)

### Prevalence of resistance to glycopeptides (vancomycin)

**Fig. 2:**
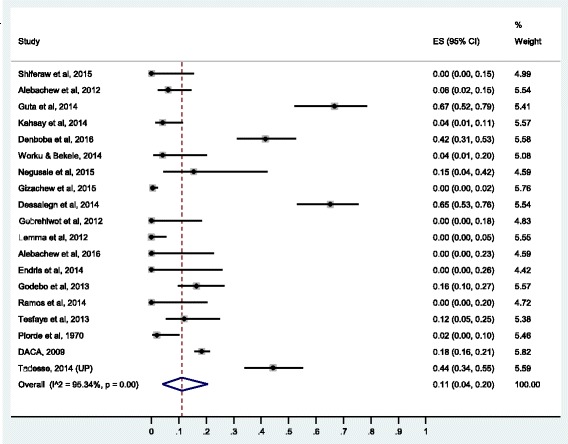
Forest plot of the prevalence of *S. aureus* resistance to vancomycin

Nineteen studies were included for meta-analysis of vancomycin resistance prevalence. The pooled prevalence for *S. aureus* resistance to vancomycin in Ethiopia is 11% (95% confidence interval [CI]: 4%, 20%). The forest plot for vancomycin resistance is presented in Fig. 2. The results of sensitivity analysis after exclusion of the two studies that appeared to report outlier prevalence values separately and both together showed non-significant influence of the two studies on the overall estimate. The pooled prevalence of vancomycin resistance when Guta et al. and Desalegn et al. were removed separately was 0.09, (95% CI: 0.03, 0.17). When, both Guta et al. and Desalegn et al. were excluded, vancomycin resistance was 0.07 (95% CI: 0.02, 0.14). All the three pooled values lie within the overall pooled estimate.

### Prevalence of resistance to penicillin’s

Here, the pooled prevalence of *S. aureus* resistance to penicillin G, amoxicillin, ampicillin, and amoxacilin-caluvanic acid was estimated. Resistance to penicillin G was estimated based on 33 studies, to amoxicillin based on 18 studies, to ampicillin based on 27 studies and to amoxacilin-caluvanic acid based on 12 studies. Pooled resistance rates were highest for β-lactamase-sensitive penicillin’s. Resistance to amoxicillin was 77% (95% CI: 68%, 85%), to penicillin G 75% (95% CI: 65%, 85%) and to ampicillin 76% (95% CI: 67%, 84%). Resistance to carbencilin (β-lactam-sensitive antibiotic) was relatively lower than other β-lactam-antibiotics (34% [95% CI: 17%, 54%]).

**Fig. 3:**
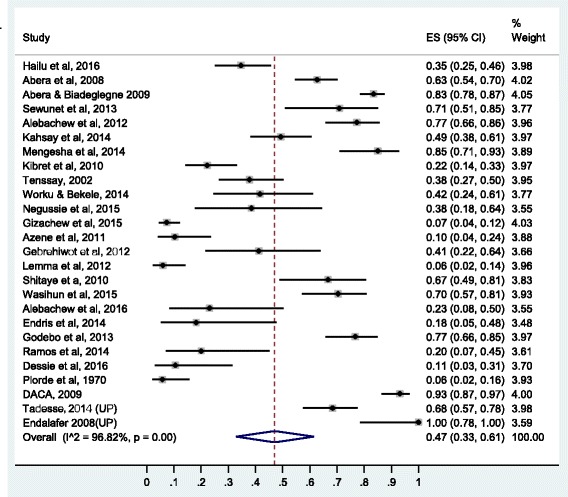
Forest plot of the prevalence of *S. aureus* resistance to methicillin

**Fig. 4:**
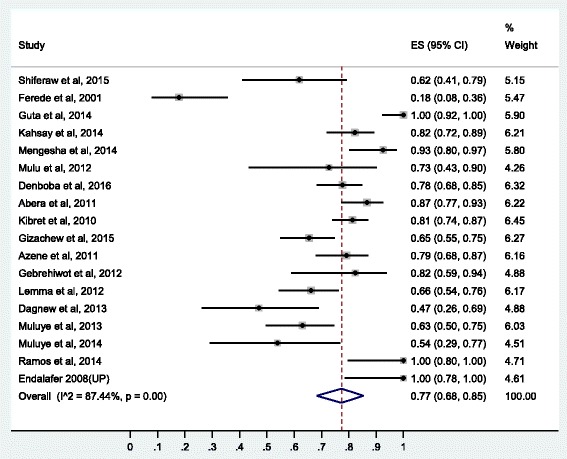
Forest plot of the prevalence of *S. aureus* resistance to amoxicillin

Relatively lower resistance rate was observed to β-lactamase-resistant penicillin’s: methicillin (47% [95% CI: 33%, 61%]) and amoxicillin-clavulanic acid (30% [95% CI: 19%, 43%]). The forest plots for methicillin and amoxacilin resistance are presented in Figs. 3 and 4, respectively while the forest plots for penicillin G, ampicillin, amoxicillin-clavulanic acid, and carbencillin resistance are presented in Additional file 3: S3, Additional file 4: S4, Additional file 5: S5 and Additional file 6: S6.

### Prevalence of resistance to cephalosporins

**Fig. 5:**
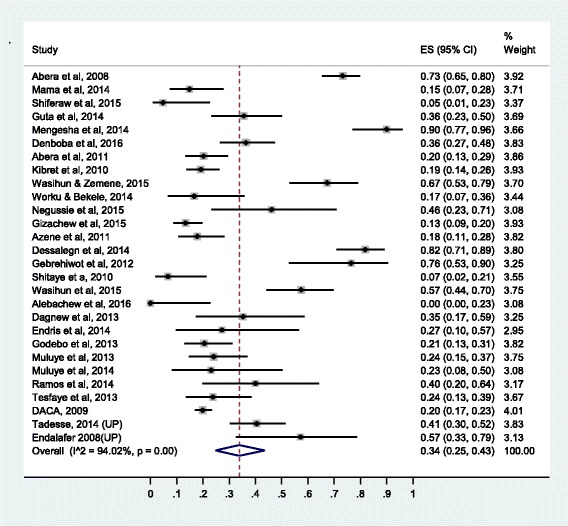
Forest plot of the prevalence of *S. aureus* resistance to ceftriaxone

Prevalence of the resistance of *S. aureus* to cephalosporins is similar to the prevalence of resistance to β-lactamase-resistant penicillin’s (amoxaclin-clavulanic acid). The prevalence of resistance to cephalothin is 30% (95% CI: 18%, 43%), to ceftriaxone 34% (95% CI: 25%, 43%) and to cefoxitine 27% (95% CI: 6%, 54%). The forest plot for ceftriaxone resistance is presented in Fig. 5 while the forest plots for cephalotine and cefoxitine resistance are presented respectively in Additional file 7: S7 and Additional file 8: S8.

### Prevalence of resistance to floroquinolones

**Fig. 6:**
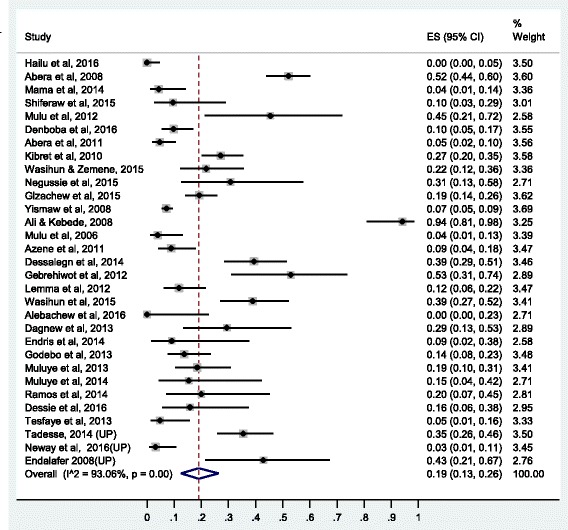
Forest plot of the prevalence of *S. aureus* resistance to ciprofloxacin

Two antimicrobial agents were tested from the floroquinolones: ciprofloxacin and norfloxacilin. Thirty one studies were used to estimate the prevalence of ciprofloxacin resistance and eleven studies were included for the estimation of norfloxacilin resistance. The pooled prevalence of *S. aureus* resistance to ciprofloxacin was 19% (95% CI: 13%, 26%) and to norfloxacillin 25% (95% CI: 14%, 38%). The forest plot for ciprofloxacin resistance is presented in Fig. 6 while the forest plot for norfloxacilin included as Additional file 9: S9.

### Prevalence of resistance to protein synthesis inhibitors

Higher rates of resistance were observed with reversible inhibitors of protein synthesis compared to aminoglycosides (irreversible inhibitors of protein synthesis). Tetracycline showed the highest resistance rate (62% [95% CI: 55%, 68%]) followed by doxycycline 43% (95% CI: 26%, 60%), erythromycin (41% [95% CI: 29%, 54%]), and chloramphenicol (37% [95% CI: 29%, 54%]). Clindamycin and aminoglycosides showed relatively lower level of resistance (Table 2).

**Fig. 7:**
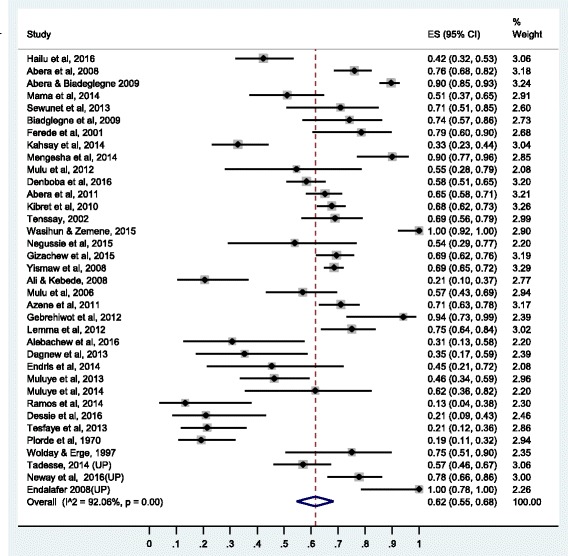
Forest plot of the prevalence of *S. aureus* resistance to tetracycline

The prevalence of resistance to gentamycin is 26% (95% CI: 18%, 34%), to amikacin 23% (95% CI: 7%, 44%) and to kanamycin 14% (95% CI: 5%, 25%). The forest plot for gentamycin resistance is presented in Fig. 7 while the forest plots for erythromycin, chloramphenicol, doxycycline, amikacin, clindamycin, and kanamycin resistance are presented as the Additional file 10: S10, Additional file 11: S11, Additional file 12: S12, Additional file 13: S13, Additional file 14: S14 and Additional file 15: S15.

### Prevalence of resistance to antimetabolites

**Fig. 8:**
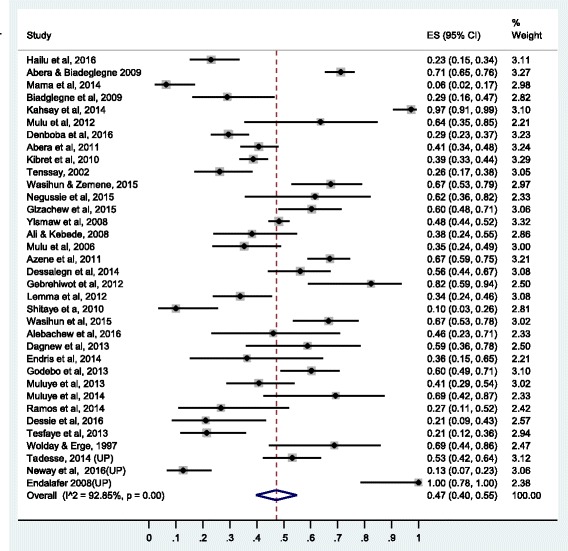
Forest plot of the prevalence of *S. aureus* resistance to sulphametaxazole-trimethoprim

Thirty five studies were included for estimation of pooled prevalence of *S. aureus* resistance to sulphametaxozole-trimethoprim and found to be 47% (95% CI: 40%, 55%). The forest plot for sulphametaxozole- trimethoprim resistance is presented in Fig. 8.

### Comparison of the prevalence of *S. aures* resistance to different antimicrobial agents

**Fig. 9:**
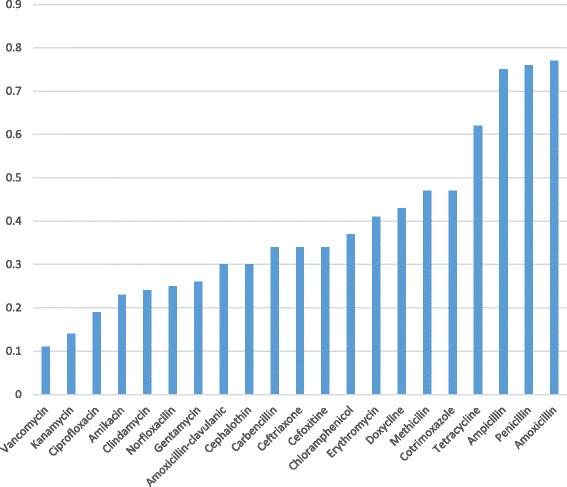
Comparison of the prevalence of *S. aureus* resistance to different antimicrobial agents in Ethiopia

Comparison of the prevalence of *S. aures* resistance to different antimicrobial agents addressed by this meta-analysis is given in Fig. 9. It is found that the magnitude of *S.aureus* resistance to the different antimicrobial agents ranges from 11% to vancomycin to 77% to amoxicillin. Accordingly, invitro antimicrobial effectiveness in decreasing order believed to be vancomycin, kanamycin, ciprofloxacilin, amikacin, clindamycin, amoxacilin-clavulanic acid, cephalothin, carbencilin, ceftriaxone, cefoxitine, chloramphenicol, erythromycin, doxycycline, methicillin, cotrimoxazole, tetracycline, ampicillin, pencilin, and amoxacilin.

## Discussion

In this meta-analysis, we estimated the pooled prevalence of *S. aureus* resistance to 21 different antimicrobial agents commonly used in Ethiopia. Generally 45 studies were included for the meta-analysis, however the number of studies included in each meta-analyses ranged from 4 to 39. Overall, the 45 studies provided evidence regarding the level of *S. aureus* resistance to different antimicrobial agents based on 4530 isolates. It was found that *S. aureus* resistance to commonly available antimicrobial agents in Ethiopia was alarmingly high ranging from 11% to vancomycin to 77% to amoxicillin.

The pooled estimate of the prevalence of *S. aureus* resistance particularly to methicillin (MRSA) in Ethiopia is similar to 2014 global surveillance reports of the World Health Organization (WHO) 2014 [88], which showed MRSA prevalence between 33% to 95% in Africa. The pooled prevalence of MRSA in Ethiopia 47% (95% CI: 33%–61%) is within the range of the global WHO report for Africa.

The pooled estimate in study for MRSA is in agreement with the pooled estimate of community acquired-MRSA prevalence in Asia, Europe, and North America which ranges from 23.1% to 47.4% [89]. However, the pooled estimate 47% (95% CI: 33%–61%) MRSA prevalence in Ethiopia is higher than pooled estimate of community acquired MRSA prevalence 30.2% based on 27 retrospective studies and 37.3% based on 5 prospective studies [90]. The higher prevalence in our study may be due to the inclusion of both community acquired and nosocomial infection in the original studies. Nosocomial infection are believed to have higher rate of resistance due to larger exposure rate to antimicrobial agents. Increasing resistance to antimicrobial agents in hospitals is caused by transmission of resistant strains within hospitals by cross colonization of patients via hands of healthcare staff and direct patient to patient contact and subsequent spread [91].

Global pattern of AMR shows variation among different geographic, socioeconomic strata and among studies [49, 88, 92]. Variation may be to differences in time, place, design, and population involved in the study. This may be due to healthcare facilities conditions like implementation and monitoring of infection prevention policies and rational antibiotic usage which varies in different facilities. The most important reason is due to character of the study. Studies are conducted within a specified time and locality. It is reasonable to assume population under study might be infected by the same strains of agent at specified period of time and location. This could be a good reason why heterogeneity tests showed significant variability (*p*-value ≤0.01) among studies included in this meta-analysis for 2 l antimicrobial agents.

*S. aureus* acquires resistance by various mechanisms: formation of alternative pathways for sulphonamides [93, 94], production of β-lactamase to β-lactam-sensitive antibiotics, increased efflux to tetracycline [95, 96], presence of acetyltransferase to chloramphenicol, decrease in accumulation to macrolide antibiotics [97], aminoglycoside-modifying enzymes production to aminoglycosides, altered topoisomerase IV and DNA gyrase expression for fluoroquinolones, and expression of mec gene altering penicillin binding protein to β-lactam antibiotics [98]. Since the AMR for β-lactam sensitive β-lactam antibiotics is very high, it can be speculated that most strains of *S. aureus* found in Ethiopia produce the β-lactamase enzyme. However, there is no molecular study conducted to identify the type of resistant strains and mechanism responsible for resistance in Ethiopia.

Lower rate of resistance was seen with β-lactamase-resistant antibiotics (amoxicillin-clavulanic acid, methicillin, ceftriaxone, cefoxtine, and cephalothin) compared to β-lactamase-sensitive penicillins. Unlike β-lactamase sensitive penicillin’s, resistance to carbeniciln is significantly lower. The lower rate of resistance observed with carbencilin and clindamycin may be due to their infrequent use in Ethiopia [99].

Resistance to methicillin confers resistance to all β-lactamase-resistant penicillins and cephalosporins. This high level of resistance requires the presence of the mec gene that encodes penicillin-binding protein [98]. The implication of high prevalence of MRSA for suspected or verified *S. aureus* infections such as common skin and wound infections and surgical prophylaxis is that there is a need for better alternatives drugs. Alternative drugs needed to treat or prevent *S. aureus* infections are more expensive and, because of their adverse effects, monitoring during treatment is advisable which increases the costs even further.

The prevalence of resistance *S. aureus* to vancomycin 11% 995% CI: (4%, 20%) in this study is bothersome and higher compared to global prevalence estimate [100]. The prevalence of VISA was 2.05% before, 2.63% in 2006–2009, and 7.93% in 2010–2014. Vancomycin resistance is erasing all possible treatment options in Ethiopia for MRSA. The higher prevalence of vancomycin in Ethiopia compared to global estimate may be due to larger and irrational use of antimicrobial agents in Ethiopia,

The prevalence estimates of glycopeptides/vancomycin resistance from Guta et al.*,* and Desalegn et al.*,* were unusually high, however sensitivity analysis showed non-significant influence on the overall pooled prevalence estimate. The prevalence estimates from Guta et al. and Desalegn et al. were unusually high, however sensitivity analysis showed non-significant influence on the overall pooled prevalence estimate. Larger exposure probability to resistant strains due to larger use of vancomycin in hospital settings might have resulted in a relatively higher prevalence of vancomycin resistance in the two studies [73, 77].

In four of the twenty studies (published in 2014 and after) [48, 51, 73, 77], the prevalence of *S. aureus* resistance to vancomycin is higher than 40%. In contrast, in studies published before 2014 the prevalence of *S. aureus* resistance to vancomycin in Ethiopia is much lower (0% to 16%). This may indicate a rapid rise and spread of vancomycin resistant *S. aureus* strains in Ethiopia as the rate of vancomycin use and exposure in Ethiopia increases. This calls for inclusion for effective new anti-MRSA antimicrobial agents for treatment of staphylococcal infections in the national medicine list and effective antimicrobial stewardship programs for prevention and containment of antimicrobial resistance.

Staphylococcal infection in Ethiopia can be better treated by vancomycin, floroquinolones, and aminoglycosides based on the finding of our invitro finding. However, clinical effectiveness study had not yet proved it. Resistance to vancomycin, the only choice for MRSA in Ethiopia, is of a great concern. It is bothersome due to lack of alternative agents in Ethiopia for the treatment of *S. aureus* infections. Making things worse, alternative new anti-MRSA agents (like linezolid, daptomycin, tigecycline, telavancin, and ceftaroline are rarely available in Ethiopia for treatment of vancomycin resistant *S. aureus.*

Many factors contribute to AMR. First, lack of infection prevention contributes to recurrent infection then to spread of resistant strains. Second, misuse of antimicrobials from prescription–dispensing-to patient use [101]. In Ethiopia, it is a common practice that antibiotics can be purchased without prescription, which leads to misuse of antibiotics by the public [102]. Third factor could be misuse of antibiotics by health professionals and non-standardized practice [101]. The fourth factor could be poor hospital hygienic conditions [103]. A last contributing factor could be lack of routine antimicrobial susceptibility testing which diverts to empiric therapy [49]. In line to strategies for prevention and containment of *S. aureus* there is a need for innovative way of halting AMR. Combination therapy and availability of new anti-MRSA agents will play vital role in fighting against AMR to *S. aureus*.

However, interpretation of the findings of this meta-analysis requires considering the limitations thereof. The limitations arise from the inherent characteristics of the included individual studies. First, this is invitro antimicrobial resistance testing and its direct translation to clinical effectiveness requires caution. Second, many studies involved very limited localities and were done mainly in teaching hospitals in bigger cities where patients with advanced, severe stages, recurrent infections are treated. Hence, the resistance level could have overestimated.

## Conclusions

This meta-analysis demonstrates that *S. aureus* has gotten alarmingly resistant to many of common antimicrobials used in Ethiopia. It is highly resistant to penicillin, cephalosporin, tetracyclines, chloramphenicol, methicillin, sulphonamides, and vancomycin. Resistance to vancomycin is of a great concern and bothersome due to unavailability of treatment options for *S. aureus* infections in Ethiopia.

Continued and multidimensional efforts of antimicrobial stewardship programme promoting rational use of antimicrobials, infection prevention and containment of AMR are urgently needed. It is deemed necessary to include new anti-MRSA agents in national medicine list to treat resistant strains. Combination therapy, effective in battling AMR in many infectious diseases model, may prove significant advantage in battling resistance to *S. aureus*. Therapeutic options are urgently needed for patients infected with resistant *S. aureus*. Further researches focusing on clinical treatment outcome and identifying dynamics promoting resistance, high risk strains and molecular genetic basis of resistance are needed.

## Additional files

Additional file 1: S1. RISMA Checklist. (DOC 63 kb)

Additional file 2: S2. Egger’s test of publication bias. (DOCX 18 kb)

Additional file 3: S3. Forest plot of the prevalence of *S. aureus* resistance to penicillin G. (DOCX 22 kb)

Additional file 4: S4. Forest plot of the prevalence of *S. aureus* resistance to Ampicillin. (DOCX 20 kb)

Additional file 5: S5. Forest plot of the prevalence of *S. aureus* resistance to amoxacilin-clavulanate. (DOCX 19 kb)

Additional file 6: S6. Forest plot of the prevalence of *S. aureus* resistance to carbencilin. (DOCX 16 kb)

Additional file 7: S7. Forest plot of the prevalence of *S. aureus* resistance to cephalothin. (DOCX 18 kb)

Additional file 8: S8. Forest plot of the prevalence of *S. aureus* resistance to cefoxitine. (DOCX 16 kb)

Additional file 9: S9. Forest plot of the prevalence of *S. aureus* resistance to norfloxacilin. (DOCX 17 kb)

Additional file 10: S10. Forest plot of the prevalence of *S. aureus* resistance to erythromycin. (DOCX 21 kb)

Additional file 11: S11. Forest plot of the prevalence of *S. aureus* resistance to chloramphenicol. (DOCX 22 kb)

Additional file 12: S12. Forest plot of the prevalence of *S. aureus* resistance to doxycycline. (DOCX 18 kb)

Additional file 13: S13. Forest plot of the prevalence of *S. aureus* resistance to amikacin. (DOCX 16 kb)

Additional file 14: S14. Forest plot of the prevalence of *S. aureus* resistance to clindamycin. (DOCX 18 kb)

Additional file 15: S15. Forest plot of the prevalence of *S. aureus* resistance to kanamycin. (DOCX 15 kb)

## Abbreviations

AMR: Antimicrobial resistance
MRSA: Methicillin resistant *Staphylococcus aureus*
NCCLs: National Committee for clinical Laboratory Standard
*S. aureus*: *Staphylococcus aureus*
VISA: Vancomycin intermediate *Staphylococcus aureus*
VRSA: Vancomycin resistant *Staphylococcus aureus*
