# Supplementary material for: Resistance of Staphylococcus aureus to antimicrobial agents in Ethiopia: a meta-analysis
Source: Antimicrob Resist Infect Control. 2017 Aug 23;6:85. doi: 10.1186/s13756-017-0243-7 (PMC5569497; doi:10.1186/s13756-017-0243-7)
Supplement: Supplementary file 2 — Egger’s test of publication bias. (DOCX 18 kb) [file 13756_2017_243_MOESM2_ESM.docx]

**Additional file S2: Egger’s test of publication bias**

|  | Antimicrobial | t (Egger's test) | *P* |
| --- | --- | --- | --- |
|  | Methicillin | -1.00 | 0.328 |
|  | Vancomycin | -0.01 | 0.994 |
|  | Ciprofloxacin | 1.52 | 0.139 |
|  | Tetracycline | -2.02 | 0.051 |
|  | Sulphametaxazole trimethoprim | -0.22 | 0.827 |
|  | Chloramphenicol | -1.08 | 0.290 |
|  | Erythromycin | -4.77 | 0.000 |
|  | Penicillin G | 0.272 | 0.272 |
|  | Clindamycin | -0.14 | 0.892 |
|  | Carbencilin | -1.06 | 0.348 |
|  | Amoxicillin | -0.68 | 0.505 |
|  | Amoxacilin-clavulanic acid | 0.13 | 0.896 |
|  | Ampicillin | 2.44 | 0.022 |
|  | Gentamycin | 0.52 | 0.605 |
|  | Ceftriaxone | 1.26 | 0.218 |
|  | Cefoxitine | 1.26 | 0.218 |
|  | Cephalothin | 0.08 | 0.936 |
|  | Doxycycline | -0.26 | 0.797 |
|  | Amkacin | -1.36 | 0.306 |
|  | Kanamycin | 0.31 | 0.771 |
|  | Norfloxacilin | 0.37 | 0.723 |
