# Supplementary material for: Resistance of Staphylococcus aureus to antimicrobial agents in Ethiopia: a meta-analysis
Source: Antimicrob Resist Infect Control. 2017 Aug 23;6:85. doi: 10.1186/s13756-017-0243-7 (PMC5569497; doi:10.1186/s13756-017-0243-7)
Supplement: Supplementary file 4 — Forest plot of the prevalence of S. aureus resistance to Ampicillin. (DOCX 20 kb) [file 13756_2017_243_MOESM4_ESM.docx]

Additional file S4: Forest plot of the prevalence of *S. aureus* resistance to Ampicillin
